# Breaching the Barrier: Investigating Initial Herpes Simplex Viral Infection and Spread in Human Skin and Mucosa

**DOI:** 10.3390/v16111790

**Published:** 2024-11-18

**Authors:** Hafsa Rana, Naomi R. Truong, Dona R. Sirimanne, Anthony L. Cunningham

**Affiliations:** 1 Centre for Virus Research, The Westmead Institute for Medical Research, Westmead, NSW 2145, Australia; hafsa.rana@sydney.edu.au (H.R.); naomi.truong@sydney.edu.au (N.R.T.); dsir3000@uni.sydney.edu.au (D.R.S.); 2 Faculty of Medicine and Health, The University of Sydney, Sydney, NSW 2006, Australia

**Keywords:** herpes simplex virus, skin, genital mucosa, viral spread, keratinocytes, dendritic cells

## Abstract

Herpes simplex virus (HSV) is sexually transmitted via the anogenital mucosa where it initially infects epidermal keratinocytes and mononuclear phagocytes (MNPs). It then spreads to the dorsal root ganglion via sensory nerve endings, to remain latent for life with periodic reactivation. Currently, there is no cure or vaccine. Initial or recurrent HSV infection can produce serious complications and mediate acquisition of HIV. This review outlines the initial events after the HSV infection of human anogenital mucosa to determine the optimal window to target the virus before it becomes latent. After infection, HSV spreads rapidly within the mid-layers of epidermal keratinocytes in the explanted human inner foreskin. Infected cells produce chemokines, which modulate nectin-1 distribution on the surface of adjacent keratinocytes, facilitating viral spread. Epidermal Langerhans cells and dendritic cells become infected with HSV followed by a “viral relay” to dermal MNPs, which then present viral antigen to T cells in the dermis or lymph nodes. These data indicate the need for interruption of spread within 24 h by diffusible vaccine-induced mediators such as antiviral cytokines from resident immune cells or antibodies. Intradermal/mucosal vaccines would need to target the relevant dermal MNPs to induce HSV-specific CD4^+^ and CD8^+^ T cells.

## 1. Epidemiology and Clinical Features of HSV1 and 2 Infections

Infection with herpes simplex virus (HSV) strains 1 and 2 is ubiquitous globally. Approximately half of the world’s population is infected with HSV1 and around 500 million people have HSV2 [1,2]. Infection with HSV can be asymptomatic or manifest in the form of skin and mucosal lesions in the orofacial region, with HSV1, and in anogenital regions, with both HSV2 and HSV1. It is one of the most common sexually transmitted infections, with HSV2 by far the leading cause of recurrent genital herpes (GH); 20% of those infected with HSV2 notice genital herpes, and asymptomatic shedding occurs in the remainder with a proportion having unnoticed lesions. HSV1 is now a common cause of initial GH, especially in adolescent and young women [3,4], but recurrence is uncommon and limited. The economic cost of GH was AUD 90 million in Australia in 2013/14 [5]. Worldwide, there are still 38 million people infected with HIV and only 21 million receiving antiretroviral therapy, resulting in only a 33% decrease in new infections. Furthermore, prior HSV2 infection increases the likelihood of HIV acquisition more than 3-fold [6,7,8]. Looker et al. estimated 30% of HIV is acquired through herpetic lesions, up to 50% in some Sub-Saharan African countries [9,10,11], and is more likely to occur soon after HSV2 acquisition [12]. Another complication of genital HSV2 is meningitis, whereas complications of orofacial HSV1 include encephalitis and keratitis, and both HSV1 and HSV2 cause severe disease in neonates [2,13,14].

In men, the main sites of initial genital HSV2 infection (and recurrence) are the inner foreskin and shaft of the penis. In women, they are the labia and vagina and, to a lesser, extent the ectocervix. Orofacial infection with HSV1 usually occurs in the mouth early in infancy or through kissing in adolescence, with secondary spread by contact to the nose or eye. Genital HSV1 infection occurs after orogenital contact [3,15,16]. Penetration of the external skin usually requires trauma, via ‘microtrauma’ in sexual intercourse [17,18], wrestling (Herpes gladiatorum) [19,20], or inflammatory skin diseases, particularly atopic dermatitis in children with the spread of herpes to the cheeks from episodes of herpes labialis [21,22]. Partners can contract HSV1 or HSV2 during the asymptomatic shedding of HSV1 into the mouth and HSV2 into the genital tract as well as during overt lesions [23,24,25].

Therefore, taking the prevalence and burden of both viruses into account, a prophylactic HSV vaccine would be likely to reduce HIV spread; vaccines for GH and HIV are WHO priorities, and their improvement is being pursued as single protein/adjuvant and RNA vaccines. A total of 30–74% efficacy against GH was achieved previously but only in seronegative women [26,27,28]. The goal of such vaccines is to prevent infection, viral shedding, or disease, in order of difficulty and preference. To prevent infection and the seeding of cutaneous nerves prior to spread to the site of lifelong latency and persistence in the dorsal root ganglia (DRG), much more needs to be understood about the initial infection and spread to the nerves at the sites of infection.

HSV1 and 2 target keratinocytes in the stratified squamous epithelium that makes up the epidermis of the skin and type II mucosal tissues such as the vagina and inner foreskin, the latter consisting of a thin layer of cornification on its surface [15,29,30,31]. The epidermis is the uppermost layer and sits atop a connective tissue layer called the dermis in skin and lamina propria in type II mucosa [32]. The inner foreskin is similar to skin, consisting of a cornified epidermis and dermis [33].

Other than the keratinocytes, HSV also infects the antigen-presenting cell populations in the epidermal layer that include Langerhans cells (LCs) and CD11c-expressing epidermal dendritic cells (Epi DCs) [34]. Both cell subsets undergo apoptosis while migrating into the dermis where dermal DC populations can take up apoptotic LCs containing viral antigen. In the primary infection, these dermal DCs can then present antigen to T cells locally and in the lymph nodes, as shown in mice, thus initiating the immune response. In recurrent infection, these dermal DCs can present antigen to resident memory T cells in the dermis [34,35,36,37]. HSV infection is restricted to the epidermis and is cleared by the combined action of multiple immune effector cells including NK cells, macrophages, and especially the combined action of CD4^+^ and CD8^+^ T cells [38,39,40,41,42,43,44,45,46,47,48,49,50]. However, HSV evades immune clearance by a rapid spread to cutaneous nerve fibres to the spinal cord and latently infecting neurons within the DRG, with subsequent periodic reactivation to the initial site of infection [51,52].

Despite the impetus provided by the global burden of illness with HSV1 and 2, there is still no cure or effective vaccine. Current treatments include antiviral medication to reduce the frequency and severity of cutaneous symptoms, but these do not clear the virus from the body [53,54]. Investigating the kinetics of HSV infection of the skin and mucosal epithelium can determine the window of opportunity to target the virus prior to it entering sensory neurons and establishing latency and evasion from the immune response. Current delays in progress to a cure or vaccine can be attributed to the difficulty of studying the disease outside of the body, especially as most individuals exhibit asymptomatic primary infection and are diagnosed late; thus, biopsy studies of human primary infection are rare [35]. Acute infection of HSV1 and 2 have been studied extensively in vitro in cell lines and artificial models of stratified squamous epithelium and in vivo using mouse models [34,35,55,56,57,58,59,60,61,62,63,64,65,66,67,68,69,70]. However, these model systems do not accurately replicate the human immune response to the infection as it first occurs. Regardless of current limitations, there have been significant discoveries in the role of inflammation, immune cells, and immunomodulatory factors during the initial infection with HSV1 and 2. This review encompasses the latest research on acute HSV1 and 2 infections of the skin and type II mucosae.

The impact of these findings lies in defining the optimal time-period to target the virus as it races against immune recognition to hide within the epidermal sensory nerve endings of the DRG neurons. This discovery will thus help guide the development of preventative vaccines and treatments against HSV.

## 2. Microtrauma Enhances HSV Entry into Skin and Mucosae

**Figure 1:**
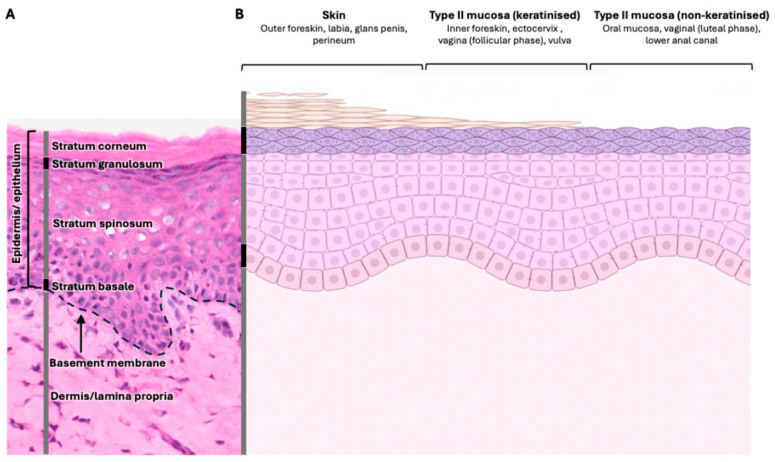
Differences in mucosal tissue types. (**A**) Haematoxylin and eosin stain of layers of skin (epidermis/dermis) and type II mucosa (epithelium/lamina propria). (**B**) Schematic diagram summarising the different locations and compositions of skin and keratinised/non-keratinised type II mucosae. Skin covers the exposed surfaces and has a thick layer of keratinization, which changes according to anatomical site. Inner foreskin is a type of type II mucosa, with keratinisation and slightly more stratification, whereas vagina and oral mucosae have a highly stratified epithelium and usually are not keratinized. Keratinisation can occur on the vagina in the follicular phase of the menstrual cycle. Created on BioRender.com.

The skin is the first point of contact with foreign pathogens and, therefore, the first line of defence. The stratified squamous epithelium that forms the epidermis of the skin consists of a series of layers (Figure 1), ~15 cells thick in humans and from superficial to deep, consisting of a cornified layer of terminally differentiated anucleated keratinocytes, or corneocytes, of the stratum corneum [71,72]. This layer provides a strong hydrophobic barrier due to the production of lipids that are deposited within the extracellular space. These lipids are secreted by the keratinocytes of the underlying stratum granulosum, which is also important in barrier function due to the presence of tight junctions [73,74]. Only once this barrier is breached and epithelial integrity is lost is cutaneous infection with HSV possible, as the virus gains access to the lower layers of the epidermis including the stratum spinosum, making up the majority of the epidermal layers, and finally the stratum basale, separated from the dermis by a basement membrane. Type II mucosa has a similar composition with variation in the barrier thickness to promote fluid exchange [31,75]. Oral and vaginal mucosa are examples of type II mucosal tissues where HSV may penetrate; however, most studies on HSV infection of these tissues were performed in animal models [76]. Facial HSV1 occurs via the oral mucosa, but recurrences occur at the lips and perinasal mucosa, which are more like surface skin [77]. There are marked similarities among oral mucosa, vaginal mucosa, and inner foreskin mucosa. All have ‘wet’ surfaces due to mucous secretion and are composed of a stratified squamous epithelium, in some cases thicker than that of general body skin, and an underlying lamina propria, and generally lack a keratinized stratum corneum [32]. The presence and thickness of keratinisation in vaginal mucosa is dependent on hormonal changes and the menstrual cycle [78]. Inner foreskin tissue has a layer of cornification and thinner epidermis than in other type II mucosal tissues [29,79].

Skin and mucosal injury and inflammation have long been considered precursors to cutaneous infection, especially in regard to sexually transmitted viruses such as HIV and HSV. Microabrasions induced by anogenital injury during anal or vaginal penetration have been shown to facilitate HIV entry. Microtrauma is common during sexual intercourse, as one study found that 55% of women and another indicated 73% of both men and women experience some level of abrasive anogenital injury [17,18,80]. In a Kenyan study, up to 66% of men experience self-reported penile injury, with an increased risk in uncircumcised men [81]. Penetrative vaginal sex leads to a pro-inflammatory environment in the female genital tract as early as one hour post intercourse [82]. This inflammatory environment, defined by increased cytokine production, cell activation, and decreased epithelial integrity (especially evident in couples with uncircumcised male partners), increases susceptibility to viral entry.

Studies showed that skin disorders, such as atopic dermatitis where epithelial integrity is reduced, promote HSV entry into the skin [66,83]. A recent study by De La Cruz et al. [66] used an in vitro model of a stratified epidermis to show that intact tight junctions prevent the HSV1 infection of keratinocytes as the virus is unable to penetrate the layer unless the barrier integrity has been disrupted. Using immunofluorescence microscopy, they showed nectin-1 expression in all layers including the stratum granulosum; however, the presence of tight junctions in this layer presumably prevents HSV access to its target receptor. Once these tight junctions are disrupted, via the promotion of an inflamed environment using IL-4 and IL-13 cytokine treatment (resembling atopic dermatitis skin), viral entry increased. Similarly, studies showed that nectin-1 is exposed in oral mucosal keratinocytes where the tight junctions were disrupted due to HIV infection, thereby increasing the rate of infection of the mucosa with HSV1 [84]. Nectin-1 is also redistributed away from cell–cell junctions in an inflammatory tissue microenvironment as a result of low extracellular calcium levels [85]. This leads to the enhanced binding of HSV glycoprotein D (gD) to nectin-1 and thus leads to a higher rate of infection [86].

Recently, Rana et al. (2024) confirmed the role of microtrauma in HSV infection using inner foreskin tissue explants, where they found that infection with HSV1 only occurred in epidermis that exhibited microabrasions to the cornified surface [59]. High-density micro-array patches (HD-MAPs) were used to deliver virus into the tissue via punctures into the skin that simulate microtears occurring during sexual transmission. Microneedles, coated with the virus, enabled access to the deeper layers of the epidermis, mainly the stratum spinosum. Viral entry did not result in infection unless the virus penetrated beyond the strata corneum and granulosum even though the epithelium was disrupted. Additionally, although rapid lateral spread was observed, infection was confined to the epidermis and did not spread to the dermis. Interestingly, another study by De La Cruz, Möckel, Wirtz et al. (2021) found that topical application after mechanical trauma via a dermaroller to human abdominal and breast skin did not induce HSV1 infection, instead resulting in minor deposits of the virus in disrupted regions of the epidermis [65]. Similarly, a study by Tajpara et al. (2019) found that puncturing abdominal skin then topically applying HSV1 did not lead to sufficient infection for their studies; however, the addition of HSV1 to the culture medium led to infection in the basal layers of the epidermis [87]. Rana et al. similarly showed that microtrauma with subsequent topical viral application did not lead to successful infection; however, coating HD-MAPs and using them as a vehicle for transfer led to the successful establishment of infection in the mid-layer, the stratum spinosum. The Rana et al. model suggests the frictional forces applied during penetration enhance viral access to target cells and closely simulates the route of infection in vivo, indicating the importance of the virus being present topically at the time of the microtrauma. Although De La Cruz et al. showed confounding results regarding the role of microtrauma in HSV infection, their work using skin explants and 3D cultures of primary keratinocytes also found that the upper strata of the epidermis are refractory to initial infection. They were only able to induce infection through the basal keratinocytes using epidermal explants submerged in HSV1 solution [65,66]. However, Rana et al. found that, in the genital inner foreskin, HSV infection could be initiated in the stratum spinosum and spread to the stratum basale. In skin, tight junction proteins are expressed throughout the epidermis, but formation of complete tight junctions is restricted to the stratum granulosum [73]. However, in vaginal mucosa, tight junctions are not as superficial as in the skin, and the inner foreskin shows a differential tight junction protein expression to the outer foreskin, presumably resulting in differences in permeability [31,75,88]. This may explain the differences in susceptibility of the stratum spinosum only in the initiation of infection.

Along with tight junction formation, the presence of antimicrobial peptides and cornified envelope proteins in the intercellular lipid matrices within the upper layers of the epidermis seems to naturally inhibit viral entry [71,83]. Cathelicidin-derived peptides such as LL-37 showed antiviral activity against HSV1 in ocular infection and in murine HSV1 and 2 infections [89,90]. RNase 7 and short-form thymic stromal lymphopoietin (sfTLSP) can also restrict the HSV1 infection of human keratinocytes [91,92,93]. The stratum granulosum was named as such due to the production of granules containing proteins such as loricrin, involucrin, and filaggrin, which eventually coat the exterior of the terminally differentiated corneocytes of the stratum corneum [71,94]. The cells of the granular layer also produce lipids such as cholesterol, free fatty acids, and sphingolipids, which surround the corneocytes and reduce water loss from the skin. It is yet to be determined whether these lipids and proteins have some refractory properties that prevent HSV infection of the granular layer, even in the presence of microtrauma. Understanding the role of these products in enhancing antiviral immunity could lead to a promising development in prophylactic treatments against cutaneous infection with HSV.

Additionally, Rana et al. [59] observed the rapid spread of HSV1 to >13 keratinocytes on average in 24 h, too fast to occur via cell-to-cell spread alone, where a single round of replication takes 12–18 h [52,95]. Indeed, single or small aggregates of HSV particles were observed in the intercellular space, supporting this additional mode of spread. Studies using HSV1-infected CHO cells and B78H1 cells found that the virus could traverse along filopodia in a manner termed as ‘surfing’. Using confocal microscopy, HSV1 was visualised moving along filopodia towards the cell body, mediated by interactions between gB and heparan sulfate found on the surface and in the extracellular matrix [70,96]. Thus, HSV1 not only spreads from cell to cell but also via the intercellular spaces possibly mediated by heparan sulfate on the cell membrane, resulting in the establishment of a rapidly spreading infection in a short period of time. The small period of time in which to target the virus prior to the spread of infection to cutaneous nerves is therefore crucial in the development of treatments or prophylactic vaccines.

## 3. HSV1 Spreads Rapidly in Keratinocytes via Nectin-1

Nectin-1 is the main entry receptor for HSV1 and 2 in keratinocyte infection via binding to gD. Nectin-1 knockdown significantly reduces HSV infection in in vitro studies [97,98], although not completely as other receptors such as the herpes virus entry mediator (HVEM) and 3-O-sulfated heparan sulfate (3-OS-HS) are still active. Nectin-1 is also expressed in all layers of the epidermis except the stratum corneum due to its primary role as an adherens junction protein, through which keratinocytes attach to one another [66]. Normally, nectin-1 can bind to itself or trans-interact with other adhesion molecules of the same family (i.e., nectin-2, -3, and -4). First, nectin-1 forms cis-homodimers, which then aggregate on the cell surface and bind a similar cluster on the adjacent cell [99,100,101,102]. The restriction of nectins to adherens junctions prevents access of gD to its receptor in healthy, intact epithelium [60,70,103,104]. HSV access to this site may also be restricted by overlying tight junctions in the skin but not genital mucosa [59,66]. During HSV infection, however, cell–cell junctions are disrupted, thus exposing the gD binding site of nectin-1. The interaction of nectin-1 with gD on the viral surface leads to a conformational change in the glycoprotein, enabling internalization of the virus with nectin-1 [70]. Thus nectin-1 appears downregulated on the cell surface during infection. Bhargava et al. (2016) found, in various cell lines, that gD directly interacts with the V domain of nectin-1 and leads to the relocalisation and subsequent internalization of this HSV receptor [70]. HSV infection then results in gD expression on the surface of the cells, which can then interact with nectin-1 on neighbouring uninfected cells to facilitate the viral spread.

These findings were only observed in vitro in cell lines until recently. Rana et al. (2024) recently reported that a similar phenomenon could be observed in situ in a human inner foreskin explant model [59]. Specifically, the absence of nectin-1 was observed in the foci of HSV1-GFP infection via immunofluorescence staining. Upon further investigation in HaCaT keratinocyte cells in vitro, the nectin-1 expression on the uninfected cells adjacent to those productively infected with HSV1 was higher. This led to the formation of a ‘collar’-like pattern of nectin-1 on the keratinocytes surrounding the focus of infection, which only occurred in the case of de novo viral synthesis as, when viral replication was inhibited, this pattern was not observed. Thus, the productive infection of the keratinocytes induced an indirect increase in accessibility to nectin-1 as a possible alternative mechanism of spread.

**Figure 2:**
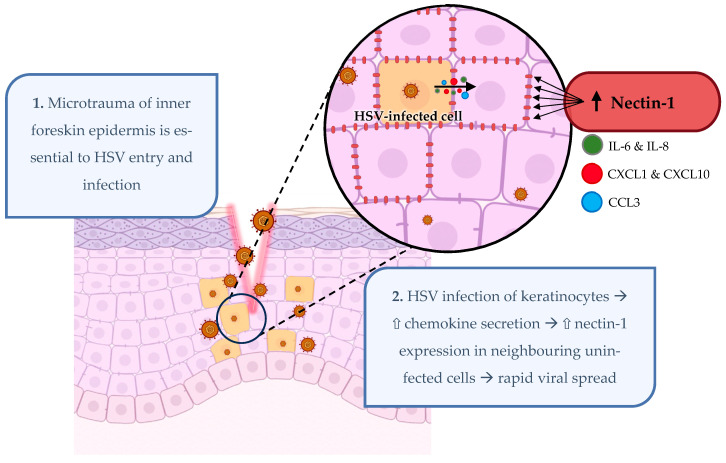
HSV infection of keratinocytes in human anogenital epidermis. Schematic diagram summarising the initial events during HSV infection of keratinocytes. Keratinocytes are infected via HSV entry receptor, nectin-1. Productive infection leads to secretion of cytokines and chemokines such as IL-6, IL-8, CXCL1, CXCL10, and CCL3. These cytokines and chemokines induce nectin-1 redistribution on neighbouring cells, promoting rapid viral spread to adjacent uninfected cells. Created on BioRender.com.

It was thus hypothesised that the productively infected keratinocytes are releasing chemomodulatory factors that lead to changes in nectin-1 in adjacent uninfected keratinocytes. The chemokines and cytokines secreted by HSV1-infected HaCaT cells were investigated via a multiplex assay, and their effects on nectin-1 localisation in keratinocytes is summarised in Figure 2. IL-8 and CCL3 were produced at significantly increased levels compared to mock infection, with trends for IL-6, CXCL1, and CXCL10, at 12 h post-infection. These cytokines also led to the redistribution of nectin-1 away from its cellular contacts when applied directly to the cells. This phenomenon was demonstrated to be at the protein level only, as quantitative PCR analysis showed the nectin-1 transcription did not change at early time points post-infection. Thus, it is evident that keratinocytes productively infected with HSV1 secrete chemokines, which act in a paracrine manner on uninfected cells, leading to the redistribution of nectin-1, exposing it to HSV1 gD, facilitating the rapid spread of the virus to the surrounding cells. There may also be other diffusible molecules acting in a paracrine fashion, which requires further investigation. Many studies showed the effect of gD on nectin-1; however, little work has been performed to investigate the cytokine production of keratinocytes as a result of HSV infection and their effect on nectin-1 localisation. Studying and controlling this mechanism of redistribution could be a key strategy in preventing the spread of HSV within the epidermis. The prospect of modulating nectins in and out of adherens junctions could be beneficial to viral infections other than HSV, such as the measles virus and polio virus [105] as well as other types of skin disorders that involve nectin-1.

## 4. Models Used to Study HSV Infection in Skin/Mucosal Systems Are Still Incomplete

The drawback to studying the biology of HSV infection of human skin/mucosa, including interactions with nectin-1, is that current models are unable to portray a holistic view of the events that take place in the tissue microenvironment. The use of human genital epidermal explant models is very informative; however, these models are subject to high donor variability and are limited in their ability to replicate a fully functional tissue environment. This is primarily due to them being closed systems that lack vasculature and therefore, lack inflammatory infiltrate. Nevertheless, the above models have contributed to the overall understanding of the tight junctions as a barrier to infection and the role of inflammatory cytokines in HSV spread. Furthermore, the period of viability for some cells in the models are short, leading to a decrease in tissue integrity over time and eventual hypoxia. In vivo murine skin models allow for a full immune infiltration over longer time points [106]. However, due to marked differences in skin thickness and MNP composition and phenotype between murine and human models, significant progress is still required to accurately mimic the in vivo response to HSV in the natural human host. This was circumvented to some degree in the past by comparing serial biopsies from human primary and/or recurrent herpes lesions with human genital explant models to examine the very early events where HSV interacts with resident epidermal immune cells [35].

Advances in microfluidics and tissue-engineering over the last decade have led to the development of in vitro “skin-on-chip” devices that aim to recapitulate human physiological responses by incorporating microvasculature [106]. Sun et al. (2022) demonstrated this by bioengineering a vascularised 3D skin-on-chip microfluidic device by first seeding primary human dermal fibroblasts within a collagen gel to form a dermal compartment and perfusing primary endothelial cells into microchannel networks fabricated within the gel matrix using soft lithography and injection moulding [107]. They then seeded keratinocytes on top of the matrix to mimic the epidermis. Similarly to HSV infection seen in vivo, the virus failed to penetrate and replicate when a cornified layer was developed on the device. The vascular nature of this device allowed for immune cell infiltration, nutrient uptake, and drug delivery. It further allowed for both live cell imaging and in situ characterisation of immune cell migration and HSV-induced cytokine secretion by keratinocytes, respectively. However, this skin-on-chip model lacks other key elements of in vivo infection that are present in the foreskin explant model, such as the absence of the skin-resident immune cells and thus an inability to accurately mimic initiation of the immune response and the full range of induced cytokine/chemokines responsible for subsequent T cell and host immune responses.

As a detailed investigation of the mechanisms of interaction of HSV and other viruses with keratinocytes is complex and difficult in stratified squamous epithelium in an in vivo or ex vivo setting, pure keratinocyte cultures have long been used and improved. However, primary human keratinocytes grown in vitro are difficult to maintain outside of their usual environment and have limited cell division capabilities [108]. Although they are isolated directly from the specific tissue and are therefore not transformed and can recapitulate tissue functions in vitro, their low expansion potential and dependence on significant amounts of fresh tissue prevent their wide use. Similar to ex vivo skin explants, this also leads to high donor variability.

The use of cell lines in studying cutaneous infection is more attractive in that they can be manipulated easily, by changing culture conditions, immune stimulation, or using gene-editing technology. Their consistency and long-term passaging capabilities provide a reliable model to study infectious diseases in skin over time. Many cell lines were used to study HSV in the past including B78H1-C10 murine melanoma cells, SY5Y cells, A431 cells, HeLa cells [69], CHO cells [109], N/TERT cells [66,110,111], and HaCaT cells [59,63,112]. The most common model cell lines are those of keratinocyte origin such as the HaCaT and N/TERT cell lines. HaCaT cells are a spontaneously immortalized keratinocyte cell line, whereas N/TERTs consist of genetically modified immortalized primary human keratinocytes [113,114]. HaCaT cells are phenotypically similar to the keratinocytes of the stratum spinosum as determined by their (albeit delayed) expression of keratins 1 and 10 [113,115]. They are mainly used in monolayer models and can differentiate due to changes in extracellular Ca^2+^ levels, cell density, and serum levels in the culture medium [113,116,117], although other studies reported that their response to changes in calcium ion concentration may be distinct from that of primary keratinocytes [115,118]. Low extracellular Ca^2+^ levels and up to 80% confluency of the cells directed them to a more basal keratinocyte phenotype, and the gradual increase in Ca^2+^ levels led to the differentiation of the cells [117]. Previous reports also showed that HaCaT cells became hyperproliferative in a higher Ca^2+^ environment, whereas primary keratinocytes were less proliferative in nature but had higher rates of differentiation, thus inducing stratification. Changes in Ca^2+^ levels also affected cell–cell junction formation [119] as high Ca^2+^ levels induced activity of cadherin proteins [120,121], which further aided the cell differentiation process. Studies by Colombo et al. (2017) and our own lab showed that HaCaT cells were able to secrete similar profiles of cytokines and chemokines as primary human keratinocytes when cultured in the appropriate conditions [59,117,122]. HaCaT cells were also used to generate organotypic skin models [123,124]; however, their transcriptional profile of cornified envelope-associated genes such as filaggrin, loricrin, and involucrin differed compared to that of primary human keratinocytes [108,118] and their ability to differentiate was limited, preventing stratum corneum formation. Thus, their stratification ability and the development of a cornified layer was impeded; however, a study by Jung et al. (2016) showed that keratinisation can be induced artificially in HaCaT 3D cultures via mechanical stress and air exposure to rescue this layer [125]. Without this artificial stimulation, however, they are not useful for studying the barrier function of the superficial epidermis on HSV1 access to nectin-1.

Another common immortalised keratinocyte cell line is N/TERT cells, which contain a transduced human telomerase reverse transcriptase (hTERT) gene and lack a key mechanism involving p16/INK4a in the cell cycle process [114]. N/TERT cells are diploid, unlike HaCaTs, which show aneuploidy, making them more likely to mutate and, therefore, less desirable in in vitro epidermal models [126]. N/TERT cells also differentiate more rapidly than primary human keratinocytes as determined by their mRNA expression of epidermal differentiation genes. They can successfully differentiate into strata granulosum and corneum layers and, when stimulated by pro-inflammatory cytokines, they show a similar increase in cornified envelope-associated gene expression as primary keratinocytes. A study by Moran, Pandya, et al. (2021) compared the barrier function, immunoreactivity, and viral infectivity of N/TERT2G cells, HaCaT cells, and primary human foreskin keratinocytes (PHFKs) [108]. Overall, they showed that N/TERT2G cells were similar in phenotype and functionality to PHFKs, unlike HaCaT cells, which showed abnormal differentiation and little to no barrier function. N/TERT cells cultured in a high Ca^2+^ concentration (1.8 mM) were able to differentiate and upregulate tight junction proteins such as occludin, claudin-1, and keratin 10 and downregulate keratin 5, which is expressed in basal keratinocytes, at a rapid rate. PHFKs in similar culture conditions expressed the same profile of proteins; however, their barrier function, measured with a trans-epithelial electrical resistance (TEER), was better than that of N/TERTs, which reached their peak TEER early and subsequently had a rapid decrease in barrier function. HaCaT cells showed differing results with a slower induction of keratin 10 and no occludin expression; thus, tight junction formation was impaired, resulting in a very low barrier function. HaCaT cells did proliferate much faster; however, they did not successfully differentiate in a similar manner to PHFKs. We can conclude that, in regard to studying the stratified squamous epidermis, N/TERT cells are most ideal, although their barrier function does not appear to be sustainable. However, in a simple monolayer of cells to study the kinetics of nectin-1 expression and HSV1 infection, HaCaT cells are still a reliable model of the mid-epidermal stratum spinosum, where the virus is most likely to initially infect keratinocytes.

Given the immortal lifespan and easily modifiable nature of HaCaT and N/TERT cell lines, they are often used in 2D and 3D models of the epidermis. Studies by De La Cruz et al. [66], among others, used N/TERT cells in 3D models of healthy and diseased epidermis to study the entry of HSV1 into keratinocytes. However, the main disadvantage to using these models to study cutaneous infections is that they are a reductionist model, which only allows the observation of interactions with a single cell type. Neither model comprises the immune cell profile or innervation of skin that is so important in the initial stages of HSV infection.

## 5. HSV Infection of Langerhans Cells, Epidermal DCs, and the Viral Relay

In addition to infecting keratinocytes within the epidermis, HSV also infects LCs and Epi DCs. Following the initial discovery of resident CD11c^+^ Epi DCs in healthy human epidermis, including their enrichment in anogenital tissues relative to LCs, and their increased uptake, replication, and transfer of HIV [127], Bertram, Truong, et al. (2021) investigated and compared their infection by HSV1 with LCs [34]. Using a GFP-tagged HSV1, Epi DCs were found to be more extensively infected with HSV1 than LCs. Furthermore, since HSV1 was shown to utilize different entry pathways in differing cell types [109,112,128,129,130], when the entry mechanisms into LCs and Epi DCs were examined, HSV1 was found to enter LCs via low pH-dependent and langerin-dependent pathways, while in Epi DCs, entry was reduced by inhibitors of actin and cholesterol, indicative of pH-independent endocytosis or macropinocytosis. Despite the differing entry pathways and increased intracellular virus in Epi DCs, during HSV infection, both cell types released only low levels of extracellular virus and became apoptotic (caspase-3^+^) by 18 h post-infection [34].

**Figure 3:**
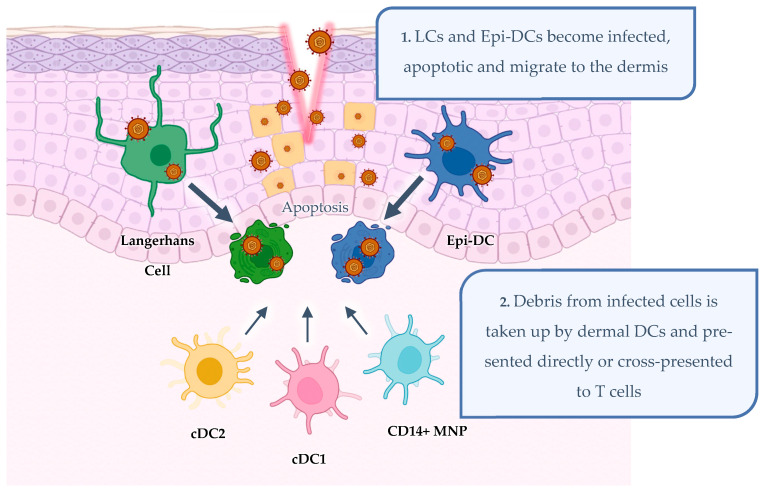
HSV infection of mononuclear phagocytes in human anogenital epidermis. HSV infects Langerhans cells and epidermal dendritic cells (Epi DCs) in the epidermis. These cells apoptose and LCs (and probably Epi-DCs) migrate to the dermis, where they cluster with dermal mononuclear phagocyte (MNP) populations including types 1 and 2 of conventional DCs (cDC1s and cDC2s) and CD14^+^ MNPs. These dermal MNPs may present to T cells at the site of infection or migrate to the lymph node for antigen presentation. Created on BioRender.com.

In a previous human study, Kim et al. (2015) also showed that LCs became apoptotic during HSV infection [35]. The transfer of HSV from apoptotic LCs to dermal DCs and macrophages was elucidated through the use of human inner foreskin explants, HSV lesion biopsies, and isolated skin MNPs and therefore was termed the “viral relay”. In this relay, HSV-infected epidermal LCs (and possibly Epi DCs, although their role in the relay remains undefined) were shown to mature and become apoptotic during migration to the dermis (24–48 h post-infection), as summarised in Figure 3. They then clustered with as many as 10 upward and laterally migrating, non-infected dermal DCs including CD14^+^ MNPs [45] and DC-SIGN^+^ macrophages at 48 h post-infection [35]. Dermal cDC1s (BDCA3^+^) and DC-SIGN^+^ macrophages phagocytosed apoptotic HSV-containing LCs with the aid of HSV-induced upregulated CLEC9A, a damaged cell uptake receptor [35,131]. CLEC9A-mediated uptake is thought to be critical in cDC1s for antigen cross-presentation through the cytosolic pathway to CD8^+^ T cells [131]. In mice, CD103^+^ dermal DCs (murine-equivalent cDC1s) were shown to emigrate during HSV infection [57] and cross-present HSV antigen to CD8^+^ T cells in lymph nodes [132]. From studies of recurrent human herpetic lesions, we know that once CD8^+^ T cells are primed, they infiltrate the site of infection, resulting in viral clearance [43]. Furthermore, HSV-specific CD8^+^ TRMs then persist at the dermo–epidermal junction near sensory nerve endings to control virus reactivation [133,134]. Thus, the stimulation of CD8^+^ T cells was shown to be critical in response to HSV infection. However, in past clinical trials, HSV vaccine candidates were unable to elicit a CD8^+^ T cell response [135]. Future vaccine approaches ought to be focussed on developing and incorporating components that can induce CD8^+^ T cell responses as well as CD4^+^ T cell responses.

Regarding CD4^+^ T cells, it has been known for decades from studies of recurrent herpes that CD4^+^ T cells infiltrate early during infection (12–48 h) [45] and their interferon (IFN)-γ production is important for combating infection in keratinocytes, reversing MHC class I downregulation [46], and for attracting CD8^+^ T cells to the site of the infection [136]. Additionally, like CD8^+^ T cells, a population of CD4^+^ T cells also persist in the upper dermis after the resolution of infection [137]. Both CD4^+^ and CD8^+^ TRMs were predicted to be critical for the rapid elimination of infection [138]. Thus, a vaccine should aim at inducing persisting CD4^+^ and CD8^+^ HSV-specific T cells as well as high levels of anti-HSV neutralising antibody and perhaps other effector mechanisms like antibody-dependent cellular cytotoxicity (ADCC) and anti-immune-evasive mechanisms.

Although Kim et al. (2015) identified DC-SIGN^+^ macrophages taking up HSV-infected LCs in the viral relay, the role of these cells in T cell priming remains to be fully elucidated. They demonstrated weak primary antigen-presenting capability but were shown to be strong stimulators of memory T cells [139]. Thus, they may play more of a role in the stimulation of memory T cells residing at the site of infection during recurrence than in primary infection. Another gap in the human viral relay is the role of dermal cDC2s. In mice, it was shown many years ago that, following HSV2 infection, rather than LCs, it was submucosal CD11c^+^ DCs that migrated to draining lymph nodes carrying viral peptides, which stimulated HSV-specific CD4^+^ T cells [140]. In human ex vivo or explant studies, distinguishing cDC2s from cDC1s requires being able to visualise more markers than the channels available by traditional fluorescence microscopes, as the traditional DC markers, such as CD1a, CD1c, and CD141 (BDCA3), can upregulate on either subset [141]. More recent studies identified CD11c, SIRPα, HLA-DQ, and FCεR1a as reliable distinguishing markers for cDC2s, whereas XCR1, CADM1, and CLEC9A are exclusive to cDC1s [141,142,143,144]. With the development of multiplexed imaging approaches, such as cyclic-immunofluorescence and imaging mass cytometry that allow larger panels of markers to be identified simultaneously, it will now be possible to identify and distinguish the roles of all known mononuclear phagocytes in the human HSV viral relay and confirm each of their T cell priming capacities.

The recruitment of other immune cell types to herpetic lesions is summarised in detail in an earlier review by Truong et al. [145]. Briefly, along with DCs and T cells, other immune subsets such as macrophages, γδ T cells, plasmacytoid DCs (pDCs), and natural killer (NK) cells were shown to play a role in the immune response to HSV infection in cell lines and mouse models, although mostly in recurrent infection. Macrophages in murine studies have dual roles in corneal HSV1 infection depending on their subtype (i.e., M1, M2). PDCs do not become infected with HSV themselves; however, direct contact leads to the production of type I interferons, among other cytokines and chemokines, which leads to the recruitment of other immune cell populations. NK cells can act directly against infected cells and kill them or indirectly via the secretion of IFN-γ, which has an antiviral effect. These cell types must be further explored in models of early infection and in biopsies to further confirm the nature of their role. Nonetheless, none of these immune cell subsets has been defined in primary herpetic lesions. The most recent study in primary herpes biopsies was by Kim et al., in 2015, where they show the uptake of HSV antigen by dermal DCs; however, they do not explore other dermal MNP populations [35].

## 6. Clinical Significance: Determining the Optimal Period for Vaccine-Induced Effectors to Work

In conclusion, although significant progress has been made using a combination of skin explants, animal models, and cell lines, ideally, the most accurate model to characterize initial HSV infection in skin will comprise the full structural and functional profile of genital skin/mucosa, including resident and infiltrating immune cells and microabrasions to the surface and functional vasculature that can be sustained over time. In the interim, cross comparisons of models of human genital skin/mucosal explants for early stages of infection, skin on a chip for late changes, and reference back to genital herpes lesion biopsies at progressive stages of the disease are the optimal way to advance the field. The lessons from the foreskin explant model developed by Rana et al. (2024) are likely to be generalisable to male and female skin and oral mucosa, in that HSV foci are initiated in the stratum spinosum and spread is not only cell to cell but intercellularly [59].

The rapid intercellular spread of HSV in genital mucosa of >13 cell diameters in 24 h would allow it to reach the vicinity of cutaneous nerve twigs and enter them soon after. These are concerning data for the feasibility of developing a vaccine to prevent infection of DRG neurons and, therefore, of subsequent recurrences of herpes. Such a vaccine would have to induce resident upper dermal memory CD4^+^ and CD8^+^ T cells able to secrete inhibitory cytokines such as IFN-γ, which would then have to induce a refractory state in keratinocytes surrounding the site of viral introduction in this period [138]. There would be no time for cellular diffusion. A diffusible antibody either from the vessels in the dermal papillae or from tissue-resident antibody-secreting B cells would probably also be necessary to interdict the intercellular spread of the virus within that 24 h period.
